# Production of low‐calorie apricot nectar sweetened with stevia: Impact on qualitative, sensory, and nutritional profiles

**DOI:** 10.1002/fsn3.1464

**Published:** 2020-03-03

**Authors:** Anna Reale, Tiziana Di Renzo, Antonio Russo, Serena Niro, Antonio Ottombrino, Mario Paolo Pellicano

**Affiliations:** ^1^ Institute of Food Science National Research Council ISA‐CNR Avellino Italy; ^2^ Department of Agricultural, Environmental and Food Sciences DiAAA University of Molise Campobasso Italy

**Keywords:** acceptability, apricot nectar, low calorie, natural sweetener, panel test, *Stevia rebaudiana bertoni*

## Abstract

This study aimed to develop a low‐calorie apricot nectar by replacing sucrose with different amount of *Stevia rebaudiana bertoni* (Rebaudioside A, 98%). Stevia has become very popular as sweetener for the production of low‐calorie products but its addition could be a challenge for industry, since it could modify sensory features of the product and consumers' acceptance. To this end, apricot nectars without sugar, with sucrose 10%, and with different amounts of stevia were produced and evaluated for microbiological quality using the pour‐plate technique, and physicochemical (pH, TTA, and *a*_w_) and nutritional (moisture, fat, protein, carbohydrates, and ash) characteristics. Furthermore, a sensory analysis of the samples was performed by a panel of trained judges using quantitative descriptive analysis. The effect of stevia addiction on the consumers' acceptance was investigated by 102 consumers of fruit juices that evaluated the overall acceptability of the samples using a structured 9‐point hedonic scale. Levels of microbial groups in nectars were under the detection limit confirming a good hygienic practice within the production. Nectars produced with stevia resulted in significant reduction in caloric value from 86 kcal (nectar with 10% sucrose) to 49 kcal (nectars with stevia), without altering its typicality. Different sensory profiles among samples were pointed out; all the products are liked, but with a different level of pleasantness. The study highlighted that the apricot nectars with 0.07% stevia are characterized for sweet and liquorice aroma notes and received the same level of consumer acceptability of nectars produced with 10% sucrose.

## Graphical abstract

*Stevia rebaudiana bertoni* extracts are a good substitute of sucrose for the production of fruit nectar. Apricot nectars with stevia are characterized for sweet and liquorice aroma notes. Nectars produced with 0.07% stevia and those produced with the addition of 10% sucrose received the same level of consumer acceptability.

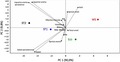

## 1 INTRODUCTION

Last researches evidenced an overarching shift toward greater consumption of sugar‐sweetened beverages (SSBs), fruit juices, nectars, and energy drinks among adolescents, in particular in low‐ to middle‐income countries (Smith, Fildes, Forwood, Cooke, & Llewellyn, 2017).

Although these beverages are extremely tasty, ready‐to‐drink and generally available at low cost, they are also characterized by a high‐calorie content, often increased by the sugar addition, generally sucrose, becoming substantial contributors to individual energy intake (Popkin & Hawkes, 2015). There have been some controversial suggestions that excessive sugar may play an important role in certain degenerative diseases like obesity (Khan & Sievenpiper, 2016), diabetes (Imamura et al., 2016), metabolic syndrome (Ferreira‐Pêgo et al., 2016), cardiovascular disease (Ross, 2015), and dental caries (Bleich & Vercammen, 2018).

As a consequence of this attentiveness, industries are replacing their old product lines or adding new products to their portfolios to supply to this segment of health‐conscious consumers with the main goal of developing healthier food products (Belc, Smeua, Macria, Vallauri, & Flynn, 2019; Gurditta, Patel, & Arora, 2019). To this end, non‐nutritive sweeteners (NNS), providing more potent sweetness and no or few calories, are increasingly popular as an alternative to sugar for the production of beverages, dietary, and dairy products (de Carvalho et al., 2019; Kalicka, Znamirowska, Pawlos, Buniowska, & Szajnar, 2019; Kumari, Arora, Choudhary, Singh, & Tomar, 2018; Lange, Scheurer, & Brauch, 2012). The United States Food and Drug Administration authority has approved six NNS (saccharine, aspartame, sucralose, neotame, acesulfame‐K, and stevia) for use in humans and has classified them under generally recognized as safe (GRAS) category (Sharma, Amarnath, Thulasimani, & Ramaswamy, 2016).

Among these, stevia has become increasingly popular as a natural sugar substitute and flavoring ingredient (Ashwell, 2015). Stevia is the generic term used to refer to different forms of the sweetener, including the whole plant stevia (*Stevia* spp.) and the leaves where the sweet compounds are found. The high sweetness of stevia is given from seven diterpenic glycosides (Ibrahim et al., 2014). Stevioside and Rebaudioside A are the main sweetening compounds, thermostable up to 200°C, making them suitable for use in cooked foods. The most important one, from a quantitative point of view, is the Stevioside that is 100–300 times more potent than sucrose, followed by Rebaudioside A, approximately 200–400 times sweeter than sucrose. Minor constituents are, instead, Rebaudiosides C, D, E and F, Dulcoside A and Steviolbioside (Ceunen & Geuns, 2013).

However, the substitution of sucrose by stevia, as sweetening agent, could be a challenge for industry, since in addition to the sweet taste, other sensory features could modify the final product. In fact, different authors (Cardello, Silva, & Damasio, 1999; Soejarto, Douglas, & Farnsworth, 1982) suggested that many Stevia species are characterized by bitter taste, probably due to sesquiterpene lactone compounds.

Miele et al. (2017) underlined that sensory science and in‐depth understanding of consumer attitude, behavior, and preference remain crucial factors in the successful development and incorporation of “healthy” products into a person's daily diet. The product reformulation may be a way of reducing sugar intake by some consumers, even though significant improvements in the sensory quality of sugar reduced products are required (Di Monaco, Miele, Cabisidan, & Cavella, 2018).

In this context, sensory analysis could be an important tool to develop novel food. Quantitative descriptive analysis is a sensory profile method and has been widely used in sensory studies of several processed foods (De Oliveira et al., 2019; Horita et al., 2017; Ng et al., 2012; Son et al., 2018; Zhang, Bowker, Yang, Pang, & Zhuang, 2020).

So, the aim of the study was to produce apricot nectar using stevia as natural sweetener to obtain a more healthy product with strongly reduced caloric index. Microbiological, physicochemical, and nutritional analyses were carried out to evaluate the qualitative characteristics of the products. Sensory profiles and the level of consumers' acceptance of the novel apricot nectars were defined.

## 2 MATERIALS AND METHODS

### 2.1 Sample fruits and nectar production process

Fresh apricots (*Prunus armeniaca* L., «Pellecchiella» variety) of good‐grade quality were harvested in a specialized farm (Masseria GioSole, Capua, CE) located in Campania Region. The fruits, collected at the right degree of ripeness (11–12° Brix), were processed within 8 hr of harvesting, transformed into purée, and then into nectar according to the manufacturer's instructions of the farm. Briefly, raw apricots were manually selected in order to eliminate fruits with evident alterations of the external surface (unripe fruits, fruits with the presence of molds etc). Fruits were firstly subjected to washing operation by turbulent flow immersion in stainless steel tanks, and subsequently by the sprinkling of running, drinking water at the exit from the tanks. After that, the fruits were pitted to remove seeds and were finely minced to obtain a puree of fruit. The nectars were produced according to the traditional recipe of the farm. In detail, puree was blended with water (60% w/w) and ascorbic acid (3 g/kg) and divided into four different batches as follows: WS, batch of sugar‐free apricot puree; S10, batch with addition of 10% of sucrose (amount of sugar used in the traditional recipe); and ST1 and ST2 batches with the addition of 0.07% and 0.14% of commercial *Stevia rebaudiana bertoni* (Rebaudioside A, 98%), respectively. Concentrations of stevia were determined considering published data on similar food matrix (Cadena et al., 2013; Cardoso & Bolini, 2008).

At the end, lemon juice was added to each batch to adjust the pH in the range 3.3–3.5. The mixture was subjected to a cooking treatment in pot for 35 min at 93°C in order to obtain the inhibition of pectinases; subsequently, it was homogenized, submitted to degassing treatment and finally filtrated on a mesh filter (ø 1 mm), filled into glass bottles (250 ml), and pasteurized at 93°C for 30 min. At last, the bottles were cooled at 35–45°C for 15–20 min and stored at room temperature. After pasteurization, the nectars were subjected to physicochemical, microbiological, and sensory analyses.

### 2.2 Microbiological analysis

Microbiological analyses were carried out within the different steps of the process as follows: fresh fruits; washed fruits; minced fruits; cooked puree; and pasteurized nectar in bottle. Samples were analyzed as described: 10 ml of sample was aseptically transferred into a sterile stomacher bag and diluted with 90 ml of physiological solution (9 g/L NaCl). After 1 min of agitation in a Stomacher 400 laboratory blender (Seward Ltd.), the samples were serially diluted and plated. Total mesophilic counts (TMC) were estimated on plate count agar after 48 hr of incubation at 28°C. Lactic acid bacteria (LAB) were counted on de Man, Rogosa, and Sharpe agar plus 4 mg/100 ml cycloheximide (SIGMA Aldrich), after incubation at 28°C for 72 hr in anaerobic conditions (Gas Pack AnaeroGen™, OXOID). Enterococci were counted on Slanetz and Bartley medium after incubation for 48 hr at 37°C. *Enterobacteriaceae* were estimated on VRBA after 36 hr at 37°C. Total and fecal coliforms were counted on VRBLA after 48 hr of incubation at 37 and 44°C, respectively. Yeasts and molds were quantified on YPD agar (bacteriological peptone 20 g/L, dextrose 20 g/L, yeast extract 10 g/L, agar 20 g/L, and streptomycin, 4 mg/100 ml). Counts were performed after 48–72 hr of incubation at 28°C. Spore‐forming bacteria were assessed on Reinforced Clostridium Medium (RCM) as described by Reale et al. (2008). All media were purchased from OXOID. Microbiological analyses were performed in triplicate.

### 2.3 Physicochemical analyses of apricot nectar

pH of nectar was measured by a pH‐meter Crison (Crison model 2001). The total titratable acidity (TTA) was determined by titrating 1 ml of sample (diluted to 20 ml final volume with deionized water) with 0.1 N NaOH. TTA values were expressed as the amount (ml) of 0.1 N NaOH necessary to achieve pH 8.3. The water activity was measured using a water activity meter (Aqualab, Decagon Devices) at a constant temperature of 23 ± 1°C. Three readings were made for each sample.

Nectars were analyzed for moisture (37.1.12 Methods of AOAC), ash (37.1.18 Methods of AOAC), carbohydrate (37.1.15 Methods of AOAC), and protein (N × 6.25) contents (37.1.35 Methods of AOAC), according to official methods of AOAC (2000). Fat has been quantified by difference to 100. Each sample was analyzed in triplicate.

### 2.4 Sensory analysis

The quantitative descriptive analysis and the acceptability tests were used to define the sensory profiles and the level of pleasantness of the different samples of apricot nectars.

#### 2.4.1 Quantitative descriptive analysis

The sensory profile of apricot nectars was obtained by applying quantitative descriptive analysis (Lawless & Heymann, 1998; Stone, Sidel, Oliver, Woolsey, & Singleton, 1974).

The sensory qualities were defined by a panel of 10 judges (five males and five females). The panel, consisting of external and internal members of the Institute of Food Science (Avellino, Italy), expert in sensory analysis, has been specifically trained for 4 weeks. In the first 3 weeks, 15 different samples of apricot nectars, purchased from supermarkets, were assessed for sensory quality (olfactory, gustatory‐tactile, retro‐olfactory, and after swallowing) during three different sessions after 1, 8, and 16 days.

At the end of each session, a round table among the judges allowed discussion about the attributes.

At the end of the 3rd week, the 14 attributes most frequently recognized by all the members of the panel were selected and the definitions and references for the maximum and minimum intensity of each attribute were determined by the trained panel (Table 1).

**Table 1:** List, definitions, and references for each sensory attribute used for the training of the judges in the sensory evaluation of apricot nectars

Sensation	Attribute	Definition	Reference
Olfactory	Olfactive intensity	Measuring the whole volume of positive and negative odors perceived through the nose	None (0); strong (10)
	Apricot smell	Evaluating the intensity of typical odor of ripe fruit	10% alcohol solution (0); 100 ppm allyl butyrate in 10% alcohol solution (10)
	Floral smell	Measuring the typical odor of flowers	10% alcohol solution (0); 1,000 ppm 2‐phenylethanol in 10% alcohol solution (10)
Taste‐tactile	Sweet	Measuring the intensity of the specific sensation of sugar	4%, 8%, and 15% sucrose solutions, intensity scale values 2, 5, and 10, respectively
	Acid	Measuring the intensity of the specific sensation caused by acidic substances	0.05% and 0.16% tartaric acid solutions, intensity scale values 2 and 8, respectively
	Bitter	Measuring the intensity of the bitterness caused by specific substances	0.06%, 0.10%, and 0.18% caffeine solutions, intensity scale values 2, 5, and 10, respectively
	Viscosity	Measuring the rate of flow across tongue of fruit juices	Water (1); condensed milk (10)
Retro‐olfactory	Apricot aroma	Evaluating the intensity of typical odor of apricot retronasally perceived	See “Apricot smell” attribute
	Liquorice aroma	Measuring the typical sensation associated with liquorice odor retronasally perceived	10% alcohol solution (0); 16 ppm estragole in 10% alcohol solution (10)
	PAI	Measuring the intensity of positive aromas retronasally perceived	None (0); strong (10)
	NAI	Measuring the intensity of negative aromas retronasally perceived	None (0); strong (10)
After swallowing	Liquorice aroma persistence	Measuring the intensity of liquorice aroma, retronasally perceived 1 min from swallowing	See liquorice aroma attribute
	Sweet persistence	Measuring the intensity of the specific sensation of sugar, 1 min from swallowing	See “Sweet” attribute
	Bitter aftertaste	Measuring the intensity of the bitterness perceived 1 min from swallowing	See bitter attribute

Note

The number in brackets indicates the intensity scale value.

Abbreviations: NAI, negative aroma intensity; PAI, positive aroma intensity.

In the 4th week of training, a quantitative assessment of the 14 selected attributes was performed in three different sessions of tasting on three apricot nectars purchased in the market. In these sessions, the judges rated 1 hr before evaluating, sweet, bitter, and acid solutions prepared as references. These three references were used to determine the performance of each judge, assessing the panelists' bias and variability, while the data obtained from tasting three apricot nectar samples were used to determine the performance of the panel for each attribute evaluating the mean and standard deviation for each member as well as for the panel as a whole. The statistical analysis demonstrated that there was good agreement among the judges. After that, the evaluation of the experimental samples started. Samples were identified with three digit numbers and evaluated twice in three different sessions. The software FIZZ Forms (Biosystemes) was used to acquire and process the data.

The sensory evaluation area was equipped with eight booths, air conditioned at 20 ± 2°C and with 50 ± 5% relative humidity, and lit with a white light at 850 Lux.

#### 2.4.2 Consumer acceptability

The consumer acceptability test was carried out in the same sensory laboratory at the same environment conditions as the quantitative descriptive analysis test. The determination of the acceptability of the different samples of nectars was accomplished by tasting test using a panel of untrained consumers. A total of 102 subjects, males and females, aged between 18 and 60 years and with the requirement to prefer and consume fruit juice or nectar, have expressed their judgment of liking through a structured 9‐point hedonic scale which comprised the following categories: 1 = Extremely unlike; 2 = Very unlike; 3 = Moderately unlike; 4 = Slightly unlike; 5 = Neither like nor unlike; 6 = Slightly like; 7 = Moderately like; 8 = Very like; and 9 = Extremely like. Approximately 20 ml of each nectar sample was served at 15°C, one at a time, and with a 5′ gap between samples. Water was provided for palate cleansing between samples.

### 2.5 Statistical analysis

Microbiological data are given as mean and standard deviation (*SD*). Physicochemical data were statistically analyzed using ANOVA analysis through SYSTAT 13.0 for Windows (Systat Software Inc.).

The quantitative descriptive analysis data were acquired and processed with the software FIZZ Forms (Biosystemes). The statistical differences among the sensory profiles of the nectar samples were evaluated by analysis of variance and the method of least significant difference (LSD). Data were subjected to principal component analysis (Granato et al., 2018). The data set consisted of a 4 × 14 matrix, in which rows represented the apricot nectar samples and columns the mean values of sensory attributes.

The acceptability of the different nectar samples was expressed as the cumulative percentage frequency of hedonic ratings of the entire panel. The differences between nectars were evaluated by Student's *t* test.

## 3 RESULTS

### 3.1 Microbial loads within the apricot nectar production

Fresh fruits were characterized by 5.36 ± 0.32 log cfu/g of TMC (Total mesophilic counts), 4.5 ± 0.23 log cfu/g of yeasts, 3.8 ± 0.19 log cfu/g of LAB (Lactic acid bacteria), 2.7 ± 0.13 log cfu/g of *Enterobacteriaceae*, 2.3 ± 0.11 log cfu/g of enterococci, and 1.8 ± 0.9 log cfu/g of spore‐forming bacteria. Total and fecal coliforms and molds were not detectable in the fresh fruits.

**Figure 1:**
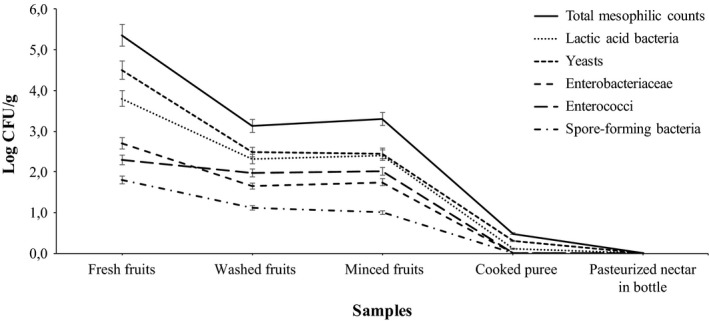
Microbiological analysis within the production of apricot nectar. Data are expressed as means ± *SD* obtained from three repeated assays

Figure 1 shows the trend of the microbial loads during the nectar production. Washing operation allowed to reduce TMC, yeasts, and LAB of about two logarithmic loads in all the samples (Washed fruits). A reduction of about one logarithmic cycle was observed for *Enterobacteriaceae*, enterococci, and spore‐forming bacteria, also. Mincing operation did not cause microbial contamination. In fact, any significant increase in microbial load was observed in the samples (minced fruits). Heating for cooking was effective in reducing all microbial counts. In fact, in the samples “Cooked puree” very few viable TMC (0.5 ± 0.19 log cfu/g) and LAB (0.1 ± 0.17 log cfu/g) counts were quantified. The pasteurization process allowed to definitely reduce all microbial loads, making the apricot nectars in the bottle microbiologically stable.

### 3.2 pH, TTA, *a*_w_, moisture, and nutritional composition

Fruit nectars from different batches had pH values ranged between 3.4 ± 0.2 (WS) and 3.6 ± 0.1 (ST2), and TTA values ranged between 3.1 ± 0.2 ml (WS) and 3.6 ± 0.7 ml (ST2) (Table 2). With respect to the pH values and TTA of the apricot nectars, there were no significant differences (*p* > .05) among the different samples, and, as also for the water activity, the values were the same in all the samples of about 0.976 (median value). Moisture values of nectars were comprised between 78.2 ± 0.21 (S10) and 87.6 ± 0.14 (WS).

**Table 2:** Physicochemical characteristics, nutritional composition, and energy values of samples of apricot nectars

Samples	pH	TTA	*a*_w_	Nutritional composition g/100 g d.w.				Moisture (%)	Energy
				Protein	Carbohydrates	Fat†	Ash		
WS	3.4 ± 0.2^a^	3.1 ± 0.2^a^	0.984 ± 0.013^a^	0.3 ± 0.01^a^	11.6 ± 0.15^a^	0.1^a^	0.4 ± 0.01^a^	87.6 ± 0.14^a^	48 kcal, 203 kJ^a^
S10	3.4 ± 0.7^a^	3.1 ± 0.2^a^	0.967 ± 0.021^a^	0.3 ± 0.03^a^	21.0 ± 0.28^b^	0.1^a^	0.4 ± 0.02^a^	78.2 ± 0.21^b^	86 kcal, 359 kJ^b^
ST1	3.5 ± 0.2^a^	3.1 ± 0.2^a^	0.974 ± 0.013^a^	0.4 ± 0.01^b^	11.6 ± 0.35^a^	0.1^a^	0.5 ± 0.02^a^	87.3 ± 0.05^a^	49 kcal, 206 kJ^a^
ST2	3.6 ± 0.1^a^	3.6 ± 0.7^a^	0.978 ± 0.011^a^	0.4 ± 0.0^b^	11.7 ± 0.15^a^	0.1^a^	0.5 ± 0.03^a^	86.1 ± 0.08^c^	49 kcal, 206 kJ^a^

Note

Different superscript letters (a, b, c) between means within a column indicate statistically significant differences (*p < .05) among different nectar samples.

Abbreviations: *a*_w_, water activity; S10, nectar with 10% sucrose; ST1, nectar with 0.07% stevia; ST2, nectar with 0.14% stevia; TTA, total titratable acidity; WS, nectar without sugar addiction.

^†^ Calculated by difference.

In Table 2 are shown the nutritional composition and energy value of the different formulations of nectars. Samples produced with stevia, ST1 and ST2, showed an ash content (about 0.5 g/100 g d.w.) similar to nectars produced without (WS) or with sucrose (S10) (about 0.4 g/100 g d.w.). Slight differences in protein content were found among the samples WS, S10, and those produced with Stevia (ST1 and ST2). Sample S10 showed the greatest value of carbohydrates (21.0 g/100 g d.w.) compared to the other samples. Very low fat values, calculated by difference, were recorded in all the samples. Sample S10 was characterized by the highest energy value (86 kcal) against 48 (WS) and 49 (ST1 and ST2) kcal.

### 3.3 Sensory analysis

**Figure 2:**
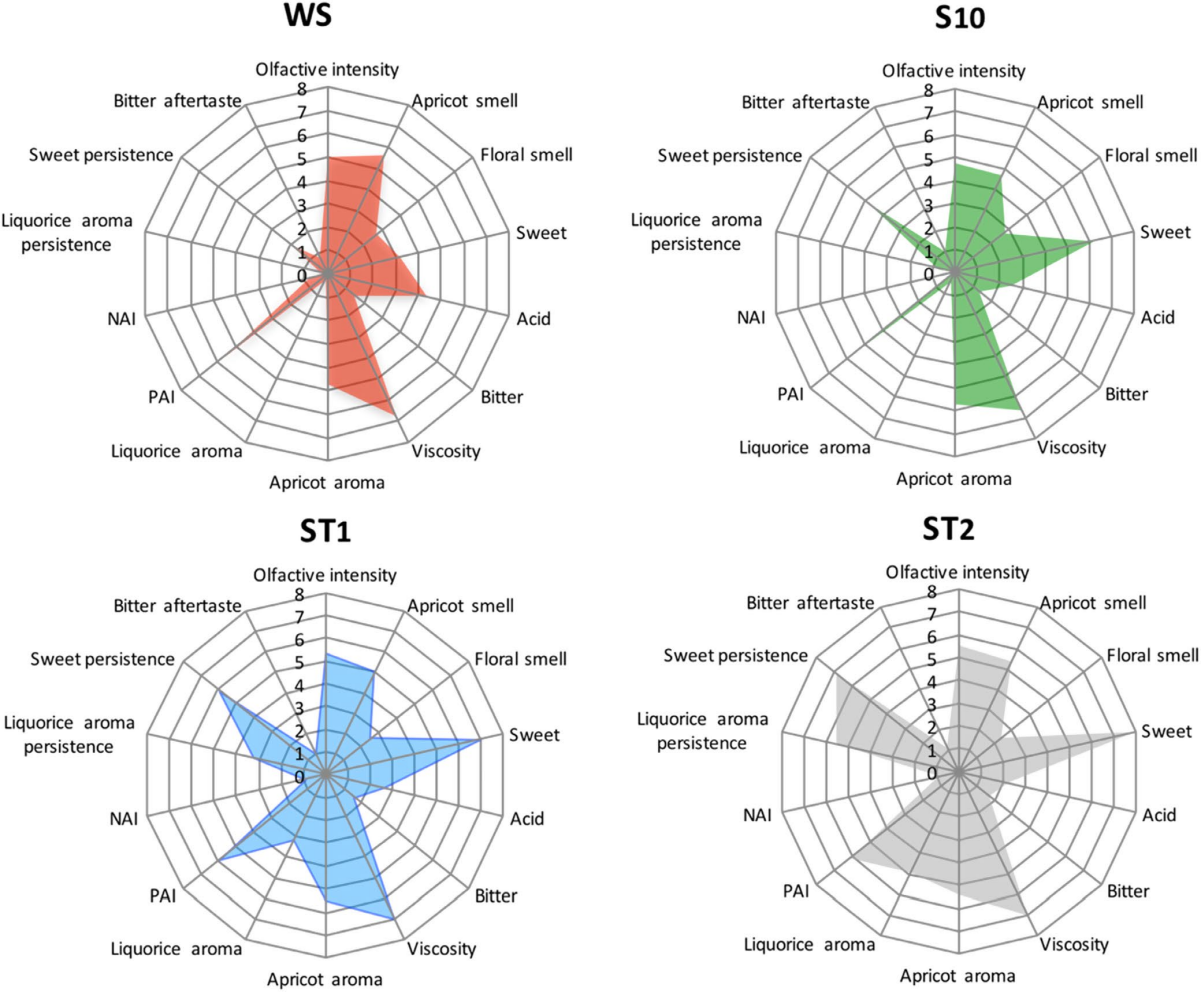
Sensory profiles of apricot nectar samples. WS, sugar‐free nectar; S10, nectar with 10% sucrose; ST1 and ST2, nectars with 0.07% and 0.14% *Stevia rebaudiana bertoni*, respectively. NAI, negative aromatic intensity; PAI, positive aromatic intensity

Figure 2 shows the sensory profiles of the different samples of apricot nectar. With regard to the attributes “Olfactive intensity,” “Apricot smell,” and “Floral smell,” no significant differences were detected among samples. Also for the attribute “Viscosity,” there were no significant differences, whereas there were significant differences for the attributes “Sweet” (*F* = 36.57, *p* < .0001) and “Acid” (*F* = 5.53, *p* < .0016).

In particular, the LSD analysis showed that the intensity of the attribute “Sweet” was significantly lower for the sample WS compared to the other three samples while the value of the sample ST2 was significantly higher with respect to the other samples. No significant differences were found between S10 and ST1 samples. For the attribute “Acid,” the determination of LSD showed that the value of the sample WS was significantly higher than the values of all the other samples, while no significant differences were found among the samples S10, ST1, and ST2.

About the retro‐olfactory sensations, significant differences among the samples were recorded only for the attribute “Liquorice aroma” (*F* = 42.48, *p* < .0001). The LSD analysis showed that the value of the sample ST2 was significantly higher than the value of the sample ST1. For the samples WS and S10, obtained without stevia addiction, the perception of “Liquorice aroma” attribute was absent. The data analysis of sensations after swallowing showed significant differences for both attributes “Liquorice aroma persistence” (*F* = 62.74, *p* < .0001) and “Sweet persistence” (*F* = 39.81, *p* < .0001). The calculation of LSD showed that for the attribute “Liquorice aroma persistence” the value of the sample ST2 was significantly higher than that of the sample ST1. For the samples WS and S10, produced without the addition of stevia, the perception of this sensation was absent. For the attribute “Sweet persistence,” all samples had values significantly different, with the sample ST2 and WS showing the highest and lowest level, respectively, and the samples ST1 and S10 comprised between them.

**Figure 3:**
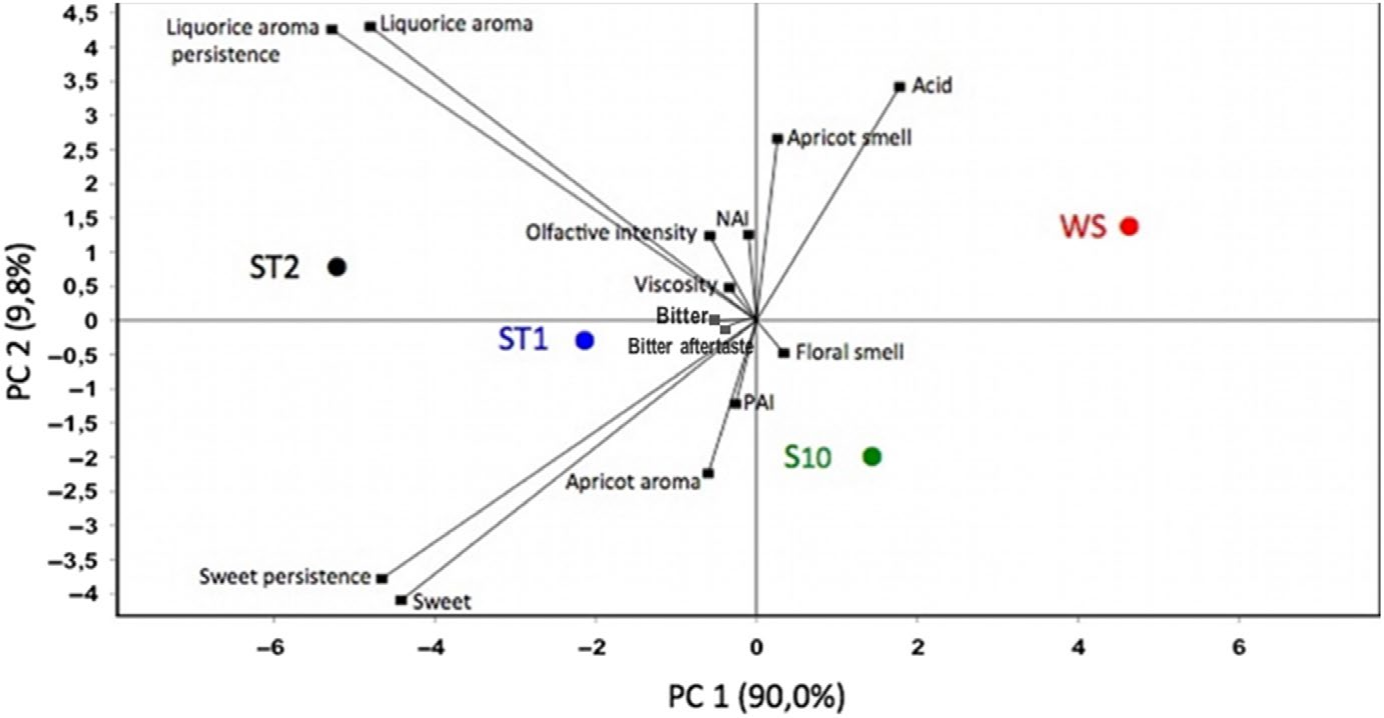
Score plot of first and second principal components after principal component analysis based on sensory attributes that mainly (*p < *.05) differentiated the apricot nectar: WS, sugar‐free apricot nectar; S10, nectar with 10% sucrose; ST1 and ST2, nectars with 0.07% and 0.14% *Stevia rebaudiana bertoni*, respectively

As shown in Figure 3, the application of principal component analysis (PCA) to the sensory data allowed to better understand the relationships between the attributes (Table 1) highlighting those that better characterized the samples of apricot nectar.

PCA distribution was strongly associated with PC1 that explained the 90% of the total variance. Attributes “Sweet,” “Sweet persistence,” “Liquorice aroma,” and “Liquorice aroma persistence” were negatively correlated with PC1, whereas “Acid” and “Apricot smell” attributes were positively correlated. All the other attributes such as “Floral smell,” “Viscosity,” “NAI,” “PAI,” “Olfactive intensity,” “Bitter,” and “Bitter aftertaste” were weakly correlated with PC1 and PC2 and were common to all the samples.

The PCA showed that the attributes “Acid” and “Apricot smell” strongly associated with the sample WS, whereas the attribute “Apricot aroma” was specific to the sample S10 and ST1. No differences among all the samples were found for the attributes “Floral smell,” “Viscosity,” “NAI,” “PAI,” “olfactive intensity,” “Bitter,” and “Bitter aftertaste.” The attributes “Liquorice aroma” and “Liquorice aroma persistence” were correlated and specific to the sample ST2 and to a lesser extent to the sample ST1. The attributes “Sweet” and “Sweet persistence” were highly correlated and common to the samples ST1, ST2, and S10.

**Figure 4:**
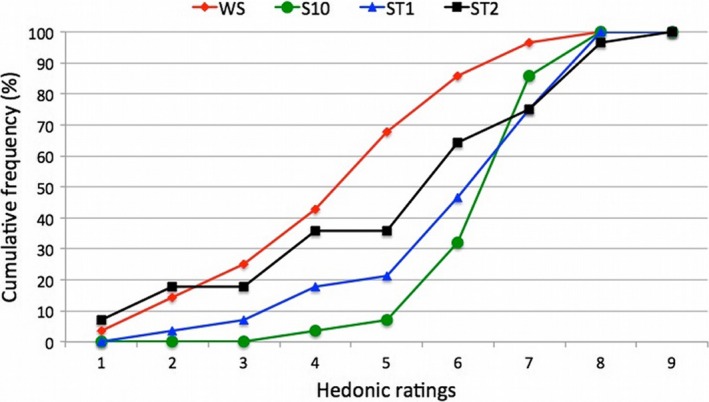
Cumulative frequency curve of hedonic ratings of the panel test of different apricot nectar samples. WS, sugar‐free apricot nectar; S10, nectar with 10% sucrose; ST1 and ST2, nectars with 0.07% and 0.14% *Stevia rebaudiana bertoni*, respectively

Figure 4 shows the acceptability expressed by consumers for the different apricot nectar samples. The results highlighted that all the products are liked, but with a different level of acceptability. In fact, sample WS received a score below 5 (neither pleasant nor unpleasant) from about 68% of consumers whereas samples ST2 and ST1 received a score below 5 from 35% and 21%, respectively. Sample S10 received a score below 5 from the lowest number of consumers (7%). Consumers rated S10 as more pleasant than both WS (*p* = .017) and ST2 (*p* = .036) and not significantly different from ST1 (*p* = .134) highlighting a same consumer's liking for the sample ST1 and S10. Also, sample WS was significantly different from ST1 (*p* = .011) and ST2 (*p* = .053). Furthermore, the data on the acceptability highlighted that the samples with stevia ST1 and ST2 were significantly different (*p* = .012) and liked with score comprised from 5‐ to 9‐point hedonic scale by 79% and by 65% of consumers panel, respectively.

## 4 DISCUSSION

### 4.1 Physicochemical, microbiological, and nutritional characteristics of apricot nectars

Raw apricots were contaminated mainly by yeasts and LAB often responsible for the fermented taste, carbon dioxide production, and production of a buttermilk off‐flavor (de Macedo, 2016; Lima Tribst, de Souza Sant'Ana, & de Massaguer, 2009; Patrignani, Tabanelli, Siroli, Gardini, & Lanciotti, 2013; Salomão, 2018; Tournas, Heeres, & Burgess, 2006). The washing operation of fresh whole fruits allowed to remove foreign material from the peel and to strongly reduce microbial contaminants. The cutting‐up and mincing operations did not let to a significant increase in load microbial levels, reflecting good hygienic standard conditions. The cooking and pasteurization operations of the apricot puree resulted in a drastic reduction of the microbial loads allowing to reach microbial loads under the detection limit and confirming the good hygienic practice on the production of fruit nectars.

Results of physicochemical properties showed that there were no significant differences in pH and TTA values between the nectars produced with stevia and those produced with or without sucrose, highlighting that the addition of stevia did not affect the acidity of the products.

About the nutritional composition, significant differences among nectars were recorded only for the carbohydrates and the energy values. Sample S10, as expected, evidenced the greatest value of carbohydrates (21.0 g/100 g d.w.) and the highest energy value (86 kcal) compared to the other samples. Instead, WS, ST1, and ST2 samples were characterized by carbohydrates and energy values not significantly different of about 11.6 g/100 g d.w. (~48 kcal).

For this reason, our data suggested that stevia added to nectars could be an opportunity to reduce the amount of sugar in the beverages consumed especially by children with high frequency, without modification of any other nutritional component. Furthermore, the European Food Safety Authority's (EFSA, 2010) Scientific Panel on additives has assessed the safety of steviol glycosides and proven that toxicological testing showed that the substances are not genotoxic, nor carcinogenic, or linked to any adverse effects on the reproductive human system or for the developing child and established an acceptable daily intake of 4 mg/kg body weight per day for their safe use.

A recent study (Boulton et al., 2016), concerning the sugar content in fruit juices consumed by children in UK, in fact, highlighted that over 40% of products surveyed contained at least 19 g of sugars—a child's entire maximum daily amount of free sugar. The authors concluded that the sugar content in products marketed to children is excessively high, and therefore, the food industries should block the addition of unnecessary sugar to packaged products.

The substitution of sugars with stevia can represent a great strategy to reduce sugar and energy intake.

Obviously, the addition of stevia could modify the sensory quality of final product; for this reason, panel test and affective test to evaluate the influence of stevia on the sensory features of the apricot nectar were carried out.

### 4.2 Sensory properties and acceptability of apricot nectars

Panel test allowed to define sensory profiles of the different apricot nectar samples. Results highlighted that samples differed mainly for taste or olfactive sensations such as sweet, acid, and liquorice attributes. In particular, S10 and even more ST1 and ST2 samples were characterized for high sweet notes and sweet persistence. On the contrary, WS sample presented the greatest intensity of acid taste. The greater intensity of acid taste in the sample WS is explained by the fact that the sensations “Sweet” and “Acid” interact at the level of the brain, in the sense that high values of the sweet sensation make the sensation of the acid feel lowest; therefore, in the WS sample without sucrose the perception of the acidic sensation was higher.

About the retro‐olfactory and after‐swallowing sensations, significant differences between the samples were recorded only for the attribute “Liquorice aroma” and “Liquorice aroma persistence.”

Moreover, differently from other studies (Cardoso & Bolini, 2008; de Melo, Bolini, & Efraim, 2009) no sensation of bitter taste was recorded after swallowing in any nectar samples produced with stevia. This result is interesting because often the major difficult of manufacturers that produce soft drink with addition of stevia is the aftertaste. This intrinsic property of stevia, as highlighted by several authors (Cardello et al., 1999), limits its application in the beverage products. In our study, we did not found a bitter aftertaste, probably for three reasons.

First of all, because the intrinsic sweet taste of the fruit and the sweet taste of the added stevia balance the sensation of bitter aftertaste induced by stevia. As evidenced by Beauchamp (2016), the suppression of bitterness by sweet and vice versa can occur at any level, from interactions between sweet and bitter molecules prior to being tasted, to interactions at the receptor protein, the receptor cell, and up to more central processes in the nervous system. As an example of central interactions between sweet and bitter taste, Kroeze and Bartoshuk (1985) used a clever split tongue psychophysical technique to show that suppression of quinine hydrochloride bitterness by sucrose was due to interactions occurring in the brain.

The second reason is that the flavor of apricot and even more the addition of lemon juice to the recipe could antagonize the bitter taste of stevia as also described by Mielby et al. (2016), which in a study highlighted that addition of lime flavor in fruit drinks was able to mask the side effect of the aftertaste caused by stevia. At last, the third reason is that the type of stevia we used (*Stevia rebaudiana bertoni*), is characterized only by high sweetness intensity and none bitter taste, as also showed by Soejarto et al. (1982) in a study carried out on 18 different species of *Stevia* spp.

Rather, the sensory properties that we highlighted among the sensation after swallowing concern the attribute “Liquorice aroma persistence,” especially in the ST2 sample that showed higher intensity, which in our opinion could influence the acceptability of apricot nectar.

The quantity of stevia added to apricot nectar seems to be of fundamental importance, since the addition of stevia 0.07% positively influences the acceptability of the products; in fact, in our study there were no significant differences in the acceptability between the sample with 10% of sucrose and that with 0.07% of stevia, whereas the quantity of 0.14% of stevia strongly decreases the acceptability of nectars. The similar acceptance behavior between apricot nectar sample with 0.07% of stevia and the one with 10% of sucrose is in agreement with what reported by Cardello et al. (1999) that highlighted that the sweet power of stevia is similar to that of a 10% sucrose solution. A similar result was reported by Reis, Alcaire, Deliza, and Ares (2017) that highlighted that regarding sensory perception of the juices with sweeteners under blind conditions, sucralose and stevia were most similar to the juice sweetened with sugar.

As observed, the sample without sugar was less accepted by the consumer, demonstrating that the consumer preferred sweet fruit nectar. Similar results have been reported by Oliveira, Ares, and Deliza (2018) that highlighted that sugar reduction in nectars caused in consumers a decrease in overall liking, which can be explained by a decrease in sweetness and an increase in sourness.

In PCA, apricot nectar samples were differently distributed in the PCA plane on the basis of specific attributes. Sample WS (upper right section of the graph), in fact, was characterized for “Acid” and “Apricot smell” attributes. Sample ST2 (upper left section of the graph) was highly associated with “Liquorice aroma” and “Liquorice aroma persistence.” Samples ST1 and S10, instead, were located near the center of the PCA plane highlighting a less specific sensory profile and highly similar characteristics. In particular, these samples were highly correlated with “Sweet,” “Sweet persistence,” and “Apricot aroma” attributes. Cumulative frequency analysis of affective study highlighted that consumers preferred samples produced with sucrose (S10) and those sweetened with stevia 0.07% (ST1), making them equivalent products from a sensory point of view but making the sample ST1 with low calorie and so healthier.

This result is relevant because, as also highlighted by other authors (Lima, Ares, & Deliza, 2019; van Raaij, Hendriksen, & Verhagen, 2009), the main challenge for reducing the added sugar content of food products and particularly in the case of sweetened beverages is that it causes changes in their sensory characteristics, which are key determinants of consumers' liking.

In fact, the consumers' perception is extremely important to the food industry since it helps to identify the factors that may impact the consumption and the purchase of new products, developing new marketing strategies (de Paula et al., 2018; Pacheco et al., 2018). Then, further projective study should be recommended for the development of new product or reformulation.

Furthermore, this study can represent a basis for undertaking investigations on the effect of stevia on qualitative and sensory properties of the apricot nectar during the shelf‐life since replacing sucrose with low‐calorie substitutes could surely influence quality of the products over time such as stated in other foods (Kaur & Goswami, 2018; Rodríguez, Magan, & Medina, 2016).

## 5 CONCLUSIONS

The proposed reformulation of apricot nectar with 0.07% stevia could be an effective strategy to reduce the added sugar content without altering the consumer acceptability. The present study confirmed that *Stevia rebaudiana bertoni*, in suitable percentages, is a good substitute of sucrose for the production of fruit nectar. The finding of our research highlighted that the apricot nectar produced with 0.07% stevia was characterized by apricot, sweet, and liquorice aroma notes and it was distinguished by the lowest calorie content alllowing to obtain a healthier fruit nectar. The novel apricot nectar received the same level of consumer acceptability compared to the sample produced with 10% sucrose encouraging its production and industrialization.

## CONFLICT OF INTEREST

All authors declare that there is no conflict of interest.

## ETHICAL APPROVAL

There was no human or animal testing in this study.
